# IL-1beta expressing neutrophil extracellular traps in *Legionella pneumophila* infection

**DOI:** 10.3389/fimmu.2025.1573151

**Published:** 2025-06-06

**Authors:** Myrto Koutantou, Theocharis Konstantinidis, Dimosthenis Chochlakis, Evangelia Xingi, Anna Psaroulaki, Georgios Tsiotis, Konstantinos Kambas, Emmanouil Angelakis

**Affiliations:** ^1^ Diagnostic Department and Public Health Laboratories, Hellenic Pasteur Institute, Athens, Greece; ^2^ Laboratory of Microbiology, School of Medicine, Democritus University of Thrace, Alexandroupolis, Greece; ^3^ Laboratory of Clinical Microbiology and Microbial Pathogenesis, School of Medicine, University of Crete, Heraklion, Greece; ^4^ Bioimaging Unit, Hellenic Pasteur Institute, Athens, Greece; ^5^ Laboratory of Biochemistry, Department of Chemistry, School of Science and Engineering, University of Crete, Heraklion, Greece; ^6^ Laboratory of Molecular Genetics, Hellenic Pasteur Institute, Athens, Greece

**Keywords:** *Legionella pneumophila*, Legionnaires’ Disease, neutrophils, neutrophil extracellular traps, NETs, IL-1beta

## Abstract

**Introduction:**

Legionella pneumophila is the causative agent of Legionnaires’ Disease (LD), an atypical pneumonia with potentially fatal outcome. Neutrophils, the first line of defense, infiltrate the lungs during L. pneumophila infection, although the precise immune mechanisms involved remain unclear.

**Methods:**

This study aims to examine in vitro the interaction of neutrophils with L. pneumophila. Neutrophils from healthy individuals were infected with opsonized and non-opsonized bacteria. Phagocytosis was assessed by immunolabeling, and reactive oxygen species (ROS) generation by flow cytometry. The ability of neutrophils to form Neutrophil Extracellular Traps (NETs) in response to L. pneumophila and the impact of these NETs on bacterial proliferation were examined. Immunolabeling and Western blotting were used for specific NET-associated epitope detection.

**Results:**

It was demonstrated that neutrophils phagocytose opsonized L. pneumophila, while non-opsonized bacteria were not phagocytosed. Opsonized bacteria triggered ROS production, unlike non-opsonized bacteria. Neutrophils released NETs upon L. pneumophila interaction in a ROS-independent manner, but these NETs failed to inhibit bacterial proliferation. Notably, IL-1b was detected on NETs.

**Discussion:**

This study provides evidence that neutrophils react to L. pneumophila through phagocytosis, the production of ROS, and NET release. IL-1b on NETs could play a role in complicated LD cases. These findings contribute to the understanding of neutrophil-mediated immune responses in LD.

## 1 Introduction

*Legionella* spp. are facultative, intracellular, Gram-negative bacteria which typically inhabit freshwater environments, and their natural hosts are amoebae and protozoa (1, 2). However, they can also multiply in poorly maintained human-made water systems, leading to human infections, with humans considered as accidental hosts (3). *Legionella* spp. can cause Pontiac fever, which is a relatively mild, self-limiting, flu-like disease, or Legionnaires’ Disease (LD) which is a more severe, life-threatening illness. The primary causative agent of LD is *Legionella pneumophila* (4). It is an important cause of Community-Acquired Pneumonia (CAP), as well as an occasional cause of Hospital-Acquired Pneumonia (HAP) (5, 6). The severity of LD ranges from a mild cough to a fatal pneumonia with an overall mortality rate of 5-10% (7). Multi-organ failure is a frequent complication correlated with a worse prognosis in LD. Research findings indicate that 61% of individuals experiencing a fatal outcome presented with complications (8).

Human infection occurs through the inhalation of contaminated aerosols, which can be generated by air conditioning systems, cooling towers, spas, fountains, showerheads, etc (9). LD is an atypical pneumonia, manifested with fever, cough, dyspnea, chills, chest pain, headache, and fatigue (6). Moreover, typical clinical manifestation of LD is diarrhea (10). In addition, extrapulmonary manifestations of LD including cardiac, brain, abdominal (gallbladder), joints, and skin involvement have been reported (11). *L. pneumophila* primarily infects alveolar macrophages and lung epithelial cells; however, it can transmigrate across the lung epithelium barrier (12), leading to organ infiltration, infection of gut epithelial cells, renal dysfunction (13–15), septic shock (15, 16), and ultimately multi-organ failure (17, 18). After invasion into the host cell, the bacterium translocates more than 300 virulence factors using its Type IV Secretion System (T4SS), in order to create a protective niche for intracellular replication (11, 19). For instance, using these effector proteins, the *L. pneumophila*-containing phagosome is transformed into an endoplasmic reticulum (ER)-like compartment called the *Legionella*-containing vacuole (LCV) (20), by hijacking protein and membrane material that are involved in vesicle trafficking (21, 22). Additionally, *L. pneumophila* employs mechanisms to inhibit autophagy (23), as well as to resist the phagosome-lysosome fusion (24, 25), thereby creating a safe replication environment.

Neutrophils are the most prevalent type of immune cells in human blood and are the first cells to migrate to the site of infection through chemotaxis during the innate immune response (26). They are a type of immune cell that play a key role in combating lung infections and have been reported to continuously patrol the lungs (27). Further, they are equipped with an impressive array of antimicrobial strategies which are tightly regulated to avoid damage in host tissues (28, 29). To counteract an infection, neutrophils use phagocytosis, degranulation, Reactive Oxygen Species (ROS) production and the release of Neutrophil Extracellular Traps (NETs) (30). Indeed, the importance of neutrophils as a first-line defense mechanism in the lungs during *L. pneumophila* infection has been previously reported (31). During pulmonary infection in mice, alveolar macrophages and neutrophils serve as the primary reservoir cells for *L. pneumophila* as well as generators of proinflammatory cytokines (32). Nonetheless, exacerbated infiltration of neutrophils has been reported as potential cause of much of the pathology associated with LD but their involvement in *Legionella* infections has not been thoroughly investigated (33, 34).

NETs are extensive web-like structures consisting of cytoplasmic, granular, and nuclear components, assembled on a scaffold of decondensed chromatin (35). NETs exhibit antimicrobial properties not only through pathogen immobilization via entrapment, but also exert a direct microbicidal activity due to antimicrobial peptides (36, 37), histones (38) and DNA (39). Interestingly, the composition of NETs varies according to the inflammatory environment in which they are formed (40) and it has been reported that NET-bound proteins remain at the site of NET formation even after the DNA backbone has been degraded (41). NETs decorated with Interleukin-1 beta (IL-1β) have been previously identified in patients with inflammatory diseases (42–44). IL-1β is a major proinflammatory cytokine which, in peripheral tissues, is essential for the effective clearance of bacterial infections (45, 46). Nevertheless, IL-1β is also involved in the development of various acute and chronic peripheral diseases (47). For instance, excessive acute activation of the IL-1β system plays a significant role in the orchestration of the cytokine storm, which can lead to severe inflammatory responses and subsequently result in a multi-organ failure associated with sepsis (48–53).

Given this context, the aim of this study was to examine *in vitro* and understand the neutrophil responses that are orchestrated in a potential *L. pneumophila* infection e.g phagocytosis, ROS generation, NET release and further identify potential mediators implicated in the pathophysiology of LD infection as specific epitopes decorating these NETs.

## 2 Materials and methods

### 2.1 Cultivation of *Legionella pneumophila*

*Legionella pneumophila* subsp. *pneumophila* (ATCC 33152^™^) was cultivated onto Buffered Charcoal-Yeast Extract (BCYE) agar plates (VWR) for 24 hours at 37°C. Bacteria were collected with a loop and resuspended into 1x Phosphate Buffered Saline (PBS) for OD_600_ measurement. Opsonization of bacteria was performed by incubation with serum from healthy donors for 30 minutes at 37°C.

### 2.2 Neutrophil and serum isolation

Peripheral blood neutrophils were isolated from heparinized blood of 10 healthy individuals (five female and five male), from whom written consent was taken, by density double-gradient centrifugation using Histopaque-1119 and Histopaque-1077 (Sigma-Aldrich) (54). After isolation, neutrophils were washed once with 1x PBS and cultured into Roswell Park Memorial Institute (RPMI 1640) medium (Gibco BRL) supplemented with 2% serum from the enrolled healthy donors (54). Neutrophil purity was ≥98% as confirmed by flow cytometry using a PE anti-human CD66b antibody (BioLegend). Healthy serum was collected from healthy blood donors with centrifugation at 1500 × *g* for 10 min at room temperature and stored in aliquots at -80°C until analysis.

### 2.3 Stimulation studies with opsonized and non-opsonized *L. pneumophila*

Neutrophils were seeded and cultured in 24-well plates at a density of 1–2 × 10^5^ cells per well in RPMI 1640 medium supplemented with 2% healthy serum, in a 5% CO_2_ atmosphere, at 37°C as previously described (42). For opsonized conditions, the cultures were supplemented with 2% healthy serum, while for non-opsonized conditions, cultures were maintained in serum-free medium. Neutrophils were incubated for 20 minutes in order to settle and were then stimulated with ionomycin as a positive control and opsonized *L. pneumophila* at a concentration of ~10 bacteria per neutrophil. For the ROS inhibition studies, neutrophils were pretreated for 15 min at 37°C with 10 μM diphenyleneiodonium chloride (DPI) (Sigma-Aldrich) before the infection. The inhibitory effect of DPI was confirmed by FACS analysis using the FagoFlowEx Kit (exbio) according to manufacturer’s recommendations with neutrophils infected by *Escherichia coli* as a positive control (**Supplementary Figure 1**). Different duration of stimulation was used depending on the assay according to optimization experiments: 60 minutes for phagocytosis assay, 30 minutes for ROS release and 3 hours for NET generation.

### 2.4 Phagocytosis determination

Phagocytosis quantification by immunofluorescence microscopy was performed as previously described (55). Peripheral blood neutrophils were seeded on poly-L-lysine coated coverslips and after stimulation, cells were fixed with 4% paraformaldehyde (PFA). Immunolabeling was conducted with the following antibodies: a rabbit anti-*L. pneumophila* polyclonal Ab (1/150; Life Technologies), a donkey anti-rabbit IgG (H+L) antibody conjugated to a fluorophore with an emission peak at 568 nm (1/500; Biotium), and DNA was counterstained by 4′,6-diamidino-2-phenylindole (DAPI) (1/10.000; Sigma-Aldrich). Images were acquired using a Leica TCS-SP8 confocal microscope with a 63x objective. Phagocytosis quantification was performed with Imaris v.9.3.1 (Oxford Instruments). Initially, the number of nuclei per image was calculated using the surfaces module. Following, a surface was created for the neutrophils (green channel - MPO), which was used as a mask for the red channel (*L. pneumophila*). In this way, Imaris generates a new channel, which contains only the *L. pneumophila* bacteria inside the neutrophils. Finally, a surface for the masked red channel was created and the number of *L. pneumophila* bacteria inside the neutrophils was calculated. Phagocytosis was quantified as the percentage of the number of neutrophils with *L. pneumophila* bacteria inside them against the total number of neutrophils (number of nuclei) per image.

### 2.5 NET visualization and quantification

NET visualization was performed by immunofluorescence microscopy as previously described (55). Peripheral blood neutrophils were seeded, stimulated and fixed as previously described. Immunolabeling was conducted with the following antibodies: a rabbit anti-citrullinated H3 (R2+R8+R17) (1/500 dilution; Abcam) polyclonal Ab, a goat anti-myeloperoxidase (MPO)-specific mAb (1/150 dilution; R&D systems) and a mouse anti-IL-1β antibody (1/200 dilution; OriGene). A donkey anti-goat IgG (H+L) antibody conjugated to a fluorophore with an emission peak at 647 nm (1/500; Biotium), a donkey anti-rabbit IgG (H+L) antibody conjugated to a fluorophore with an emission peak at 568 nm (1/500; Biotium), and a donkey anti-mouse IgG (H+L) antibody conjugated to a fluorophore with an emission peak at 488 nm (1/500; Biotium) were utilized as secondary antibodies. DNA was counterstained by DAPI (1/10.000; Sigma-Aldrich). Images were acquired using a Leica TCS-SP8 confocal microscope with a 20x objective. The percentage of NET-releasing cells was determined by eye examination of several fields from every sample. Quantification of NET release was conducted with a MPO/DNA complex ELISA as previously demonstrated (56, 57). Briefly, 50 μl of a 5 μg/ml anti-MPO mAb was coated onto 96-well plates overnight at 4°C. Following washing, 20 μl of samples and 80 μl incubation buffer with peroxidase-labeled anti-DNA mAb were added. The plate was incubated for 2 hours at room temperature with shaking at 300 rpm. After washing, 100 μl ABTS peroxidase substrate was added, and absorbance at 405 nm was measured after 20 minutes in the dark. NET formation was expressed as the percentage increase in absorbance above the control.

### 2.6 ROS measurement

Measurement of respiratory burst of neutrophils after incubation with different stimuli was performed using the FagoFlowEx Kit (exbio) according to the manufacturer’s instructions with modifications as follows. Purified neutrophils from the healthy individuals were incubated in the presence of dihydrorhodamine 123 (DHR123), for 30 minutes, at 37°C, with non-opsonized as well as with opsonized *L. pneumophila.* After incubation, cells were lysed, and ROS were determined using a flow cytometer (BD FACSCalibur).

### 2.7 NET structures isolation and co-cultivation with *L. pneumophila*

Peripheral blood neutrophils were seeded in 6-well culture plates at a density of 1 × 10^6^ cells per well in RPMI 1640 medium, supplemented with 2% healthy serum, for 20 minutes in order to settle and were then incubated with ionomycin as a positive control and with opsonized *L. pneumophila* bacteria for another 3 hours (37°C, 5% CO_2_). After incubation, the supernatant was removed, and cells were washed with fresh medium. NET structures were collected after vigorous agitation. The medium was centrifuged at 50 x *g* for 5 minutes at 4°C to remove debris and NETs were collected in supernatant phase (58). For the co-cultivation assay assessing the ability of NETs to inhibit *L. pneumophila* proliferation, heat-inactivated *L. pneumophila* was used to trigger NET release in neutrophils in order to avoid pre-existing alive bacteria on the NET structures. Extracellular DNA formations were collected and 300μl of each condition was incubated with freshly prepared *L. pneumophila* bacteria in a ratio of 3:1 for 30 minutes, at 37°C, and after incubation directly plated onto prewarmed BCYE agar plates for the examination of the ability of ionomycin-induced and *L. pneumophila*-induced NETs to retain *L. pneumophila* growth.

### 2.8 NET protein isolation for biochemical assays

To isolate the protein load of the produced NETs, supernatants were removed following incubation with the stimuli for 3 hours in the 6-well plates as mentioned previously, and cells were washed with fresh medium once. DNA was digested with micrococcal nuclease (10 U/ml; Thermo Fisher) for 20 min at 37°C in the presence of 50 mM Tris-HCl pH 8.0 and 5 mM CaCl_2_. Supernatants were collected and centrifuged at 300 x *g* for 5 min at 4°C to remove debris and supernatants containing the protein load were transferred to a fresh tube for storage at −80°C until further analysis (59).

### 2.9 Western Blot

An automated Jess Simple Western Blot system (ProteinSimple, USA) was used according to the manufacturer’s standard method for 12-230-kDa Jess separation module (SMW004). The same volume from each NET protein sample was mixed with 0.1X Sample buffer and Fluorescent 5X Master mix (ProteinSimple) in the presence of fluorescent molecular weight markers and 400 mM dithiothreitol (ProteinSimple) and was denatured at 95°C for 5 min. The ladder and the protein samples were separated in capillaries as they migrated through a separation matrix. After separation, proteins were immobilized by photoactivated capture chemistry on the capillaries. A 1/1000 dilution of the mouse anti- IL-1β antibody and a RTU anti-mouse secondary antibody (ProteinSimple) were utilized for the identification of the target. Peroxyde/luminol-S (ProteinSimple) was used to establish the chemiluminescent revelation. The Compass Simple Western software (Protein Simple) was utilized to capture a digital image of the capillary’s chemiluminescence. The software automatically determined the area, signal/noise ratio, and heights (chemiluminescence intensity).

### 2.10 Ethics approval statement

All study participants provided written informed consent in accordance with the principles expressed in the Declaration of Helsinki. Participants’ records were anonymized and de-identified prior to analysis to ensure anonymity and confidentiality. The study protocol was approved by the Ethics Committee of the Hellenic Pasteur Institute (Approval Number 3702/12 - 06- 2024).

## 3 Results

### 3.1 Neutrophils phagocytose *L. pneumophila* following opsonization

**Figure 1:**
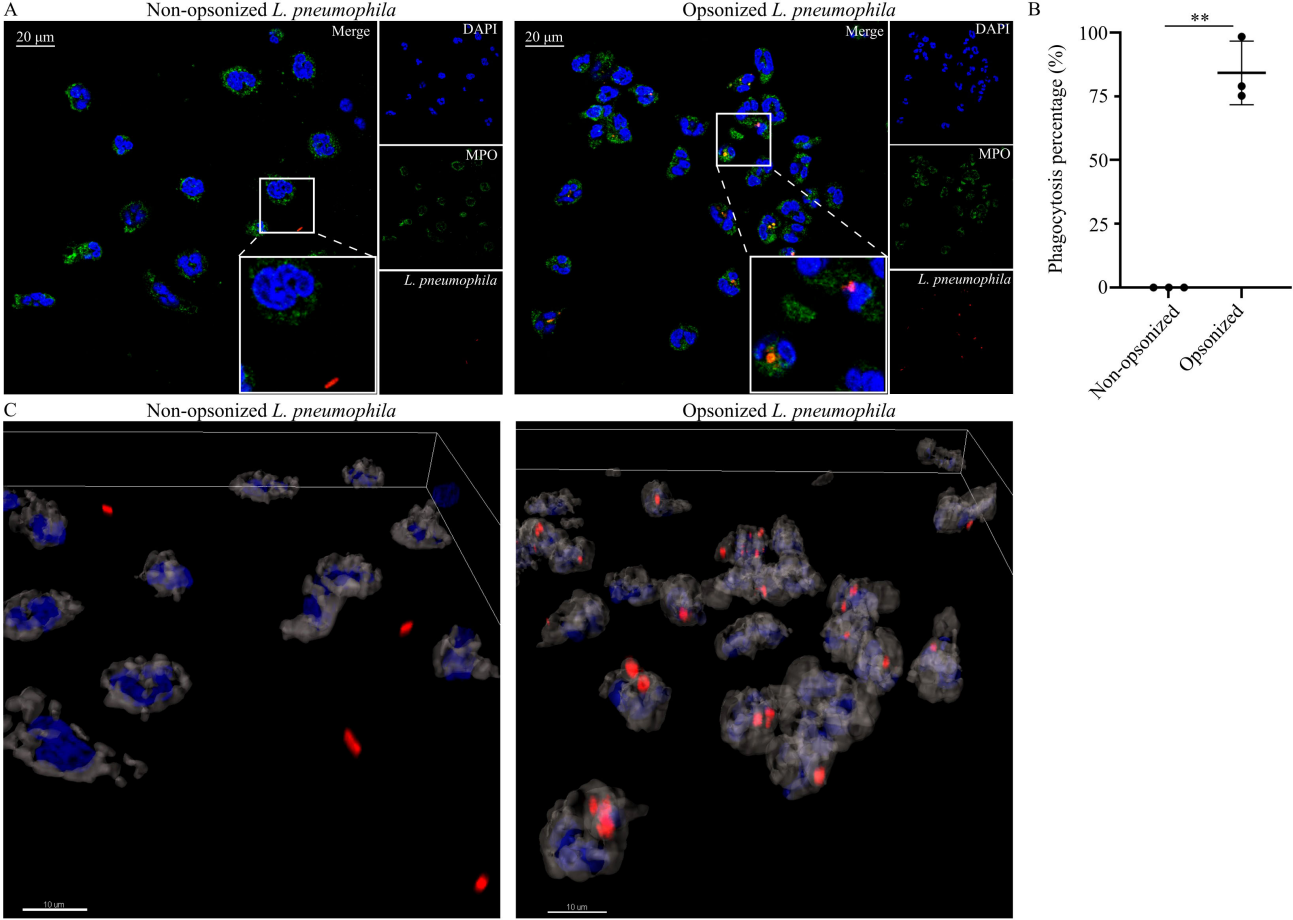
Neutrophils phagocytose opsonized *L. pneumophila*. **(A)** HC neutrophils cocultured with either non-opsonized (serum-free conditions) *L. pneumophila* (mean ± SD; 0.0% ± 0, n=3) or serum-opsonized *L. pneumophila* (mean ± SD; 84.19% ± 12.45, n=3. P = 0.0072), (confocal microscopy; blue: DAPI/DNA, green: MPO, red: *L. pneumophila*). Images were acquired with a Leica TCS SP8 confocal microscope using a 63x objective. Scale bar, 20 μm. **(B)** Quantification of phagocytosis using Imaris v 9.3.1. Data from 3 independent experiments in panel **(B)** are presented as means ± SDs (**p <0.01). **(C)** 3D reconstruction of neutrophils infected with opsonized and non-opsonized *L. pneumophila*. Scale bar, 10 μm.

One of the mechanisms that neutrophils use to eliminate pathogens is phagocytosis. Hence, we investigated whether neutrophils are able to phagocytose *L. pneumophila* in *in vitro* conditions. Image analysis by confocal microscopy revealed that healthy control (HC) neutrophils were able to phagocytose serum-opsonized *L. pneumophila* bacteria, when the latter were added in HC cultures (**Figure 1A**). In contrast, phagocytosis was absent in non-opsonized *L. pneumophila* bacteria (**Figure 1A**). Quantification of phagocytosis was performed using Imaris v 9.3.1 and was defined as the percentage of neutrophils with *L. pneumophila* bacteria inside them against the total number of neutrophils (**Figure 1B**). Additionally, 3D models were constructed using Imaris v 9.3.1 which clearly show that in opsonized conditions, *L. pneumophila* are inside the neutrophils in contrast to non-opsonized conditions (**Figure 1C**). These findings demonstrate the ability of neutrophils to phagocytose *in vitro L. pneumophila* after serum opsonization.

### 3.2 Neutrophils produce ROS in a phagocytosis-dependent manner in response to *L. pneumophila*

**Figure 2:**
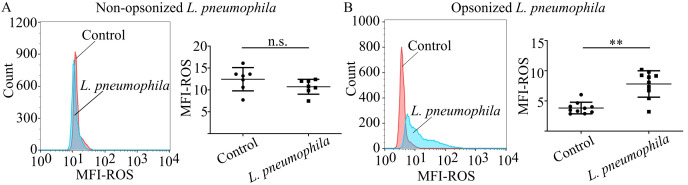
Neutrophils produce ROS in response to *L. pneumophila* in a phagocytosis-dependent manner. ROS production in neutrophils from **(A)** HC neutrophils (mean ROS MFI ± SD; 12.45 ± 2.654, n=7) cocultured with non-opsonized *L. pneumophila* in serum-free conditions (mean ROS MFI ± SD; 10.72 ± 1.703, n=7. p = 0.0903) and **(B)** HC neutrophils (mean ROS MFI ± SD; 3.835 ± 0.9617, n=10) cocultured with opsonized *L. pneumophila* (mean ROS MFI ± SD; 7.819 ± 2.190, n=10. p = 0.002). Data from 7 independent experiments in panel **(A)** and 10 independent experiments in panel **(B)** are presented as means ± SDs (n.s., not significant, p >0.05; **p <0.01).

It has been previously shown that neutrophils are able to produce ROS as another defense mechanism in response to *L. pneumophila* (60). However, the implication of phagocytosis in the generation of ROS was not investigated. To assess the role of phagocytosis in ROS generation in neutrophils by *L. pneumophila*, HC neutrophils were cocultured with *L. pneumophila* in the presence or absence of opsonization conditions. In the presence of opsonization, coculturing with *L. pneumophila* indicated a statistically significant increase of ROS generation compared to HC neutrophils, as demonstrated by flow cytometry (**Figure 2B**). In the absence of opsonization, *L. pneumophila* did not induce ROS generation (**Figure 2A**) compared to controls. These findings indicate that ROS generation is induced in neutrophils by *L. pneumophila* bacteria in a highly phagocytosis-dependent manner since non-opsonized bacteria lose the capacity to induce ROS.

### 3.3 Neutrophils release NETs as a response to *L. pneumophila*

**Figure 3:**
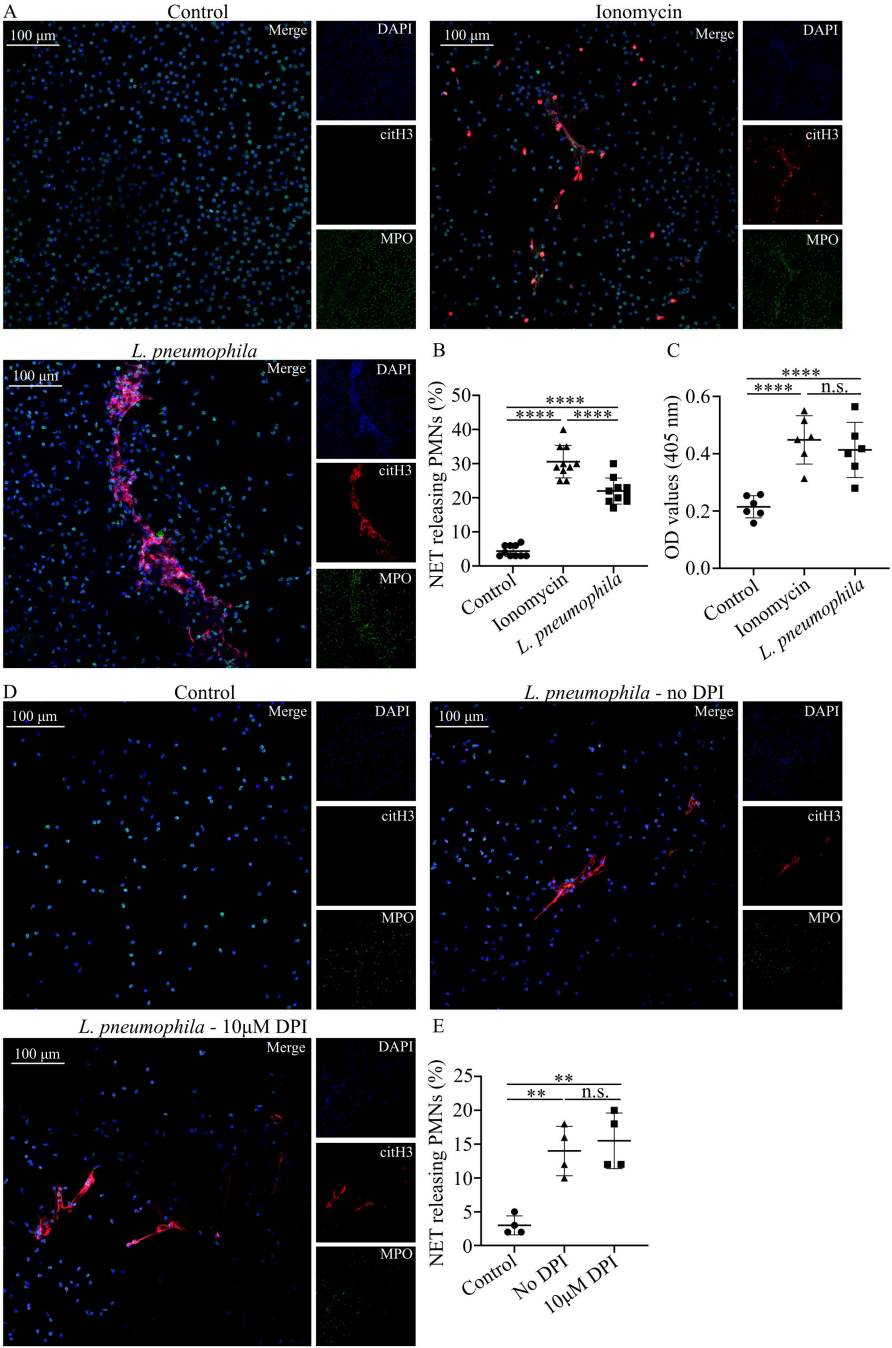
*L. pneumophila* induces NETs in neutrophils *in vitro* in a ROS-independent manner. **(A)** NETs in HC peripheral blood neutrophils (mean ± SD; 4.4% ± 1.647, n=10), either treated with ionomycin (mean ± SD; 30.6% ± 4.742, n=10, p < 0.0001) or cocultured with *L. pneumophila* (mean ± SD; 22% ± 3.801, n=10. p < 0.0001) (confocal microscopy; blue: DAPI/DNA, green: MPO, red: citH3). Images were acquired with a Leica TCS SP8 confocal microscope using a 20x objective. Scale bar, 100 μm. **(B)** Quantification of NET-releasing neutrophils from **(A)** and **(C)** Quantification of NET releasing neutrophils with MPO/DNA complex ELISA. **(D)** NETs in HC peripheral blood neutrophils cocultured with *L. pneumophila* in the presence (mean ± SD; 15.5% ± 4.123, n=4. p > 0.9999) or absence (mean ± SD; 14% ± 3.651, n=4.) of DPI. Images were acquired with a Leica TCS SP8 confocal microscope using a 20x objective. Scale bar, 100 μm. **(E)** Quantification of NET-releasing neutrophils from **(D)** Data from 10 independent experiments in panel **(B)**, from 6 independent experiments in panel **(C)** and 4 independent experiments in panel **(E)** are presented as means ± SDs (n.s., not significant, p >0.05; **p <0.01; ****p <0.0001).

Taking into account the ability of neutrophils to generate NETs as a defense mechanism in a potential infection (28, 30), we investigated whether *L. pneumophila* infection could potentially induce this defense mechanism. Coculturing of HC neutrophils with opsonized *L. pneumophila* indicated statistically significant increased NETs release compared to controls (**Figure 3B**), as observed by immunofluorescence (**Figure 3A**) and quantified by MPO/DNA complex ELISA (**Figure 3C**). HC neutrophils cocultured with *L. pneumophila* demonstrated lower levels of NETs release compared to ionomycin treated HC neutrophils (**Figure 3B**).

Previous reports demonstrated that NET release triggered by Gram-negative pathogens is ROS-dependent (61, 62). Since *L. pneumophila* has the capacity to induce ROS to HC neutrophils we investigated if the observed increase in NETs release is ROS-dependent. HC neutrophils pre-treated with DPI before the addition of *L. pneumophila* demonstrated similar NET release to HC treated with *L. pneumophila* alone (**Figures 3D, E**). This finding indicates that NET release by *L. pneumophila* is ROS-independent.

### 3.4 NETs are unable to restrain *L. pneumophila* growth *in vitro*

**Figure 4:**
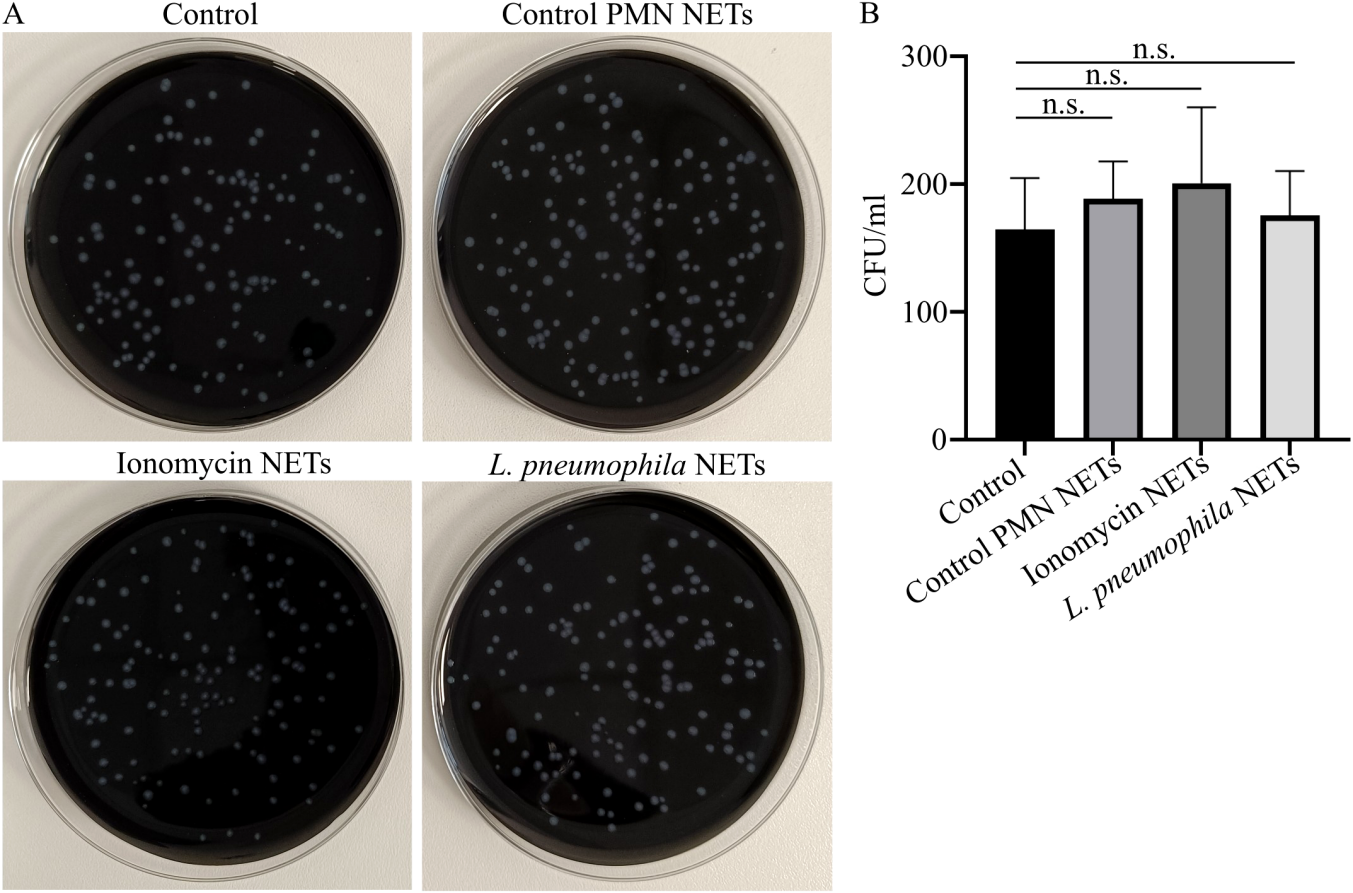
*L. pneumophila* proliferation is not inhibited in the presence of NET structures. **(A)** *L. pneumophila* cultures in BCYE plates (mean CFU/ml ± SD; 195.3 ± 80.17, n=4.) in the presence or absence of NETs induced by different stimuli; ionomycin (mean CFU/ml ± SD; 211.3 ± 74.27, n=4. p = 0.9909) or *L. pneumophila* (mean CFU/ml ± SD; 202.8 ± 72.41, n=4. p = 0.9990) or control NETs (vehicle from untreated neutrophils) and **(B)** Quantification of CFU from **(A)**. In panel **(B)**, data from 4 independent experiments are presented as means ± SDs (n.s., not significant, p >0.05).

Given the bactericidal properties of NETs (30) and the ability of neutrophils to release NETs in response to *L. pneumophila*, we examined whether these NET structures can inhibit *L. pneumophila* growth *in vitro. L. pneumophila*-induced NET structures *in vitro* did not have an effect on the growth of *L. pneumophila* compared to control conditions, as observed macroscopically (**Figures 4A, B**). Similarly, ionomycin-induced NET structures had no significant effect. This finding indicates that *L. pneumophila* growth is not inhibited by NETs.

### 3.5 Identification of IL-1β on NETs induced by opsonized *L. pneumophila*

**Figure 5:**
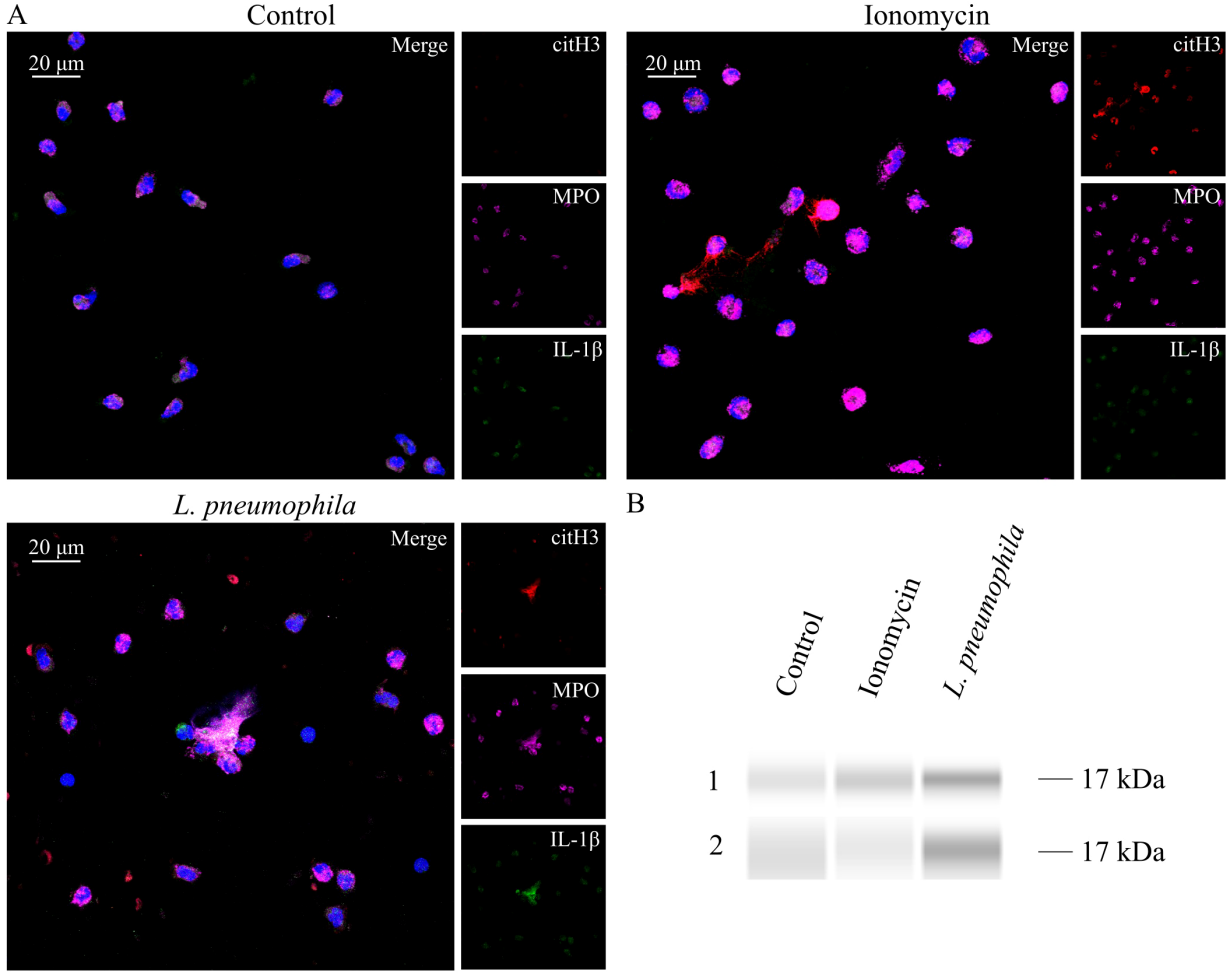
*L. pneumophila* induces IL-1β expressing NETs *in vitro*. **(A)** NETs in HC peripheral blood neutrophils, either treated with ionomycin or cocultured with *L. pneumophila* (confocal microscopy; blue: DAPI/DNA, red: citH3, magenta: MPO, green: IL−1β). Images were acquired with a Leica TCS SP8 confocal microscope using a 20x objective. Scale bar, 20 μm. **(B)** Western Blot analysis of NET proteins derived from healthy controls after treatment with ionomycin or coculture with *L. pneumophila*.

IL-1β is a major proinflammatory cytokine that plays a pivotal role in initiating and regulating the cytokine storm, which can ultimately result in multi-organ failure (51). Considering that multi-organ failure is a common complication of LD and that NETs can express bioactive IL-1β under inflammatory conditions (43, 44, 63, 64), we investigated the presence of IL-1β on NETs induced by *L. pneumophila*. NETs generated *in vitro* by HC neutrophils in response to *L. pneumophila* were decorated with IL-1β as observed by confocal microscopy (**Figure 5A**) and confirmed by immunoblotting (**Figure 5B**). On the other hand, IL-1β was not prevalent on ionomycin-induced NETs. This finding suggests that IL-1β is expressed on NETs induced by *L. pneumophila in vitro*.

## 4 Discussion

In this report we demonstrated for the first time that HC neutrophils infected *in vitro* with opsonized *L. pneumophila*, produce NETs decorated with IL-1β. Although neutrophils have been reported to release NETs decorated with IL-1β in sterile inflammation (43, 44, 63, 64), the expression of IL-1β on NETs due to an infecting agent has not been reported previously. Moreover, we demonstrated that this NETs release is not ROS-dependent and *L. pneumophila* growth is unaffected by NETs.

Neutrophils are able to restrict *L. pneumophila* infection as previously demonstrated (60, 65, 66), however the mechanism of the immune response has only partially been explored. In agreement with the findings of others, we also observed that opsonized *L. pneumophila* bacteria were significantly phagocytosed by neutrophils, but non-opsonized *L. pneumophila* were phagocytosed only to a small extend (60, 67). Also, in accordance with previous studies (60, 66), we demonstrated that neutrophils produce ROS in response to *L. pneumophila*. However, we further determined that ROS production was phagocytosis-dependent, since the infection with opsonized *L. pneumophila* was resulting in significantly more ROS than non-opsonized bacteria. Moreover, we provide evidence for the first time that opsonized *L. pneumophila* induced significant NET release *in vitro* in HC neutrophils. To our knowledge, there is no previous research on the ability of *L. pneumophila* to induce NET release, nor the role of NETs in host defense against *L. pneumophila* infection. Several other pneumonia-causing bacteria, such as *Streptococcus pneumoniae* (68), *Staphylococcus aureus* (69), *Haemophilus influenzae* (70), *Klebsiella pneumoniae* (35), and *Mycoplasma tuberculosis* (71) have been previously studied and are also known to induce NET release (30). Additionally, we observed that NET release was ROS-independent, since NET release was not affected after the inhibition of ROS. Initially, it was thought that only ROS-dependent NET release was possible (62), however, Pilsczek et al. reported a novel NET release mechanism in response to *S. aureus* that was ROS-independent (69). *Leishmania* parasites have also been found to trigger ROS-independent NET release (72).

Functional studies regarding the NET structures induced by opsonized *L. pneumophila* as well as by ionomycin demonstrated that these NETs were unable to restrain *L. pneumophila* growth *in vitro*. Bactericidal properties of NETs alone have also been questioned in the case of *S. aureus* (73). Likewise, several pneumonia-causing bacteria are known to have evolved mechanisms that escape NETs, like *S. pneumoniae* (68), *S. aureus* (74), *H. influenzae* (70) and *M. tuberculosis* (71).

In certain instances, NETs not only fail to benefit the host, but instead they may contribute significantly to the pathophysiology of various diseases (75). For example, it has been demonstrated that in sepsis patients, NETs decorated with Tissue Factor are implicated in the initiation of the coagulation cascade, which is a critical step in disseminated intravascular coagulation and ultimately lead to multi-organ failure (76). Additionally, the excessive release of NETs has been linked to multiple organ dysfunction in sepsis, as inhibiting NET release led to diminished organ dysfunction and reduced lethality in septic mice (77). Specifically, the protein load of NETs has also been linked to non-infectious pathologies (78–82). IL-1β was previously identified as a decorative component of NETs in patients with inflammatory diseases like active ulcerative colitis (63) and non-alcoholic steatohepatitis (43). Additionally, IL-1β has been identified on NETs of Familial Mediterranean Fever patients during their attack episodes (44, 64). Interestingly, in Familial Mediterranean Fever patients, circulating levels of IL-1β are not correlated to the severity of the disease, however, they respond highly to IL-1β blockade therapy, indicating its fundamental role in the orchestration of the disease (83, 84).

The ability of IL-1β blockade therapy to prevent multi-organ failure has been previously evaluated. In sepsis patients, in the subgroup of multi-organ dysfunction syndrome, where the inflammasome pathway is implicated, anakinra had been shown to have beneficial effects (85). Additionally, a subgroup of patients with COVID-19 was linked to increased levels of IL-1β, severe cytokine storm and hyperinflammatory symptoms (86, 87). Several studies have investigated the role of anakinra treatment on COVID-19 revealing beneficial results (48, 88–91).

This study’s main limitation is the absence of clinical specimens in order to observe NETs *ex vivo* or *in situ* in patient biopsies. This leads to the lack of direct clinical evidence. Furthermore, another limitation is the lack of functional assays of IL-1β on *L. pneumophila* induced NETs which would clarify its contribution to the disease. Further studies are needed to expand our findings and improve our understanding of the pathophysiology of LD.

In conclusion, we provide evidence that upon *L. pneumophila* infection, neutrophils fight bacteria by phagocytosis, generation of ROS in a phagocytosis-dependent manner and release of NETs. These findings contribute significantly to the understanding of the effect of neutrophils in neutralizing a *L. pneumophila* infection. In addition, the presence of NETs decorated with IL-1β in response to *L. pneumophila* may provide some insight into the pathophysiology of multi-organ failure in LD and could trigger further investigation into its association with the disease.

## Data Availability

The original contributions presented in the study are included in the article/**Supplementary Material**. Further inquiries can be directed to the corresponding authors.

## Supplementary material

Supplementary Figure 1 HC neutrophils infected with opsonized *E. coli* bacteria in the absence and presence of 10 μM or 100 μM DPI. Data shown are representative of three independent experiments.
