# Supplementary figures and images for: IL-1beta expressing neutrophil extracellular traps in Legionella pneumophila infection
Source: Front Immunol. 2025 Jun 6;16:1573151. doi: 10.3389/fimmu.2025.1573151 (PMC12179218; doi:10.3389/fimmu.2025.1573151)

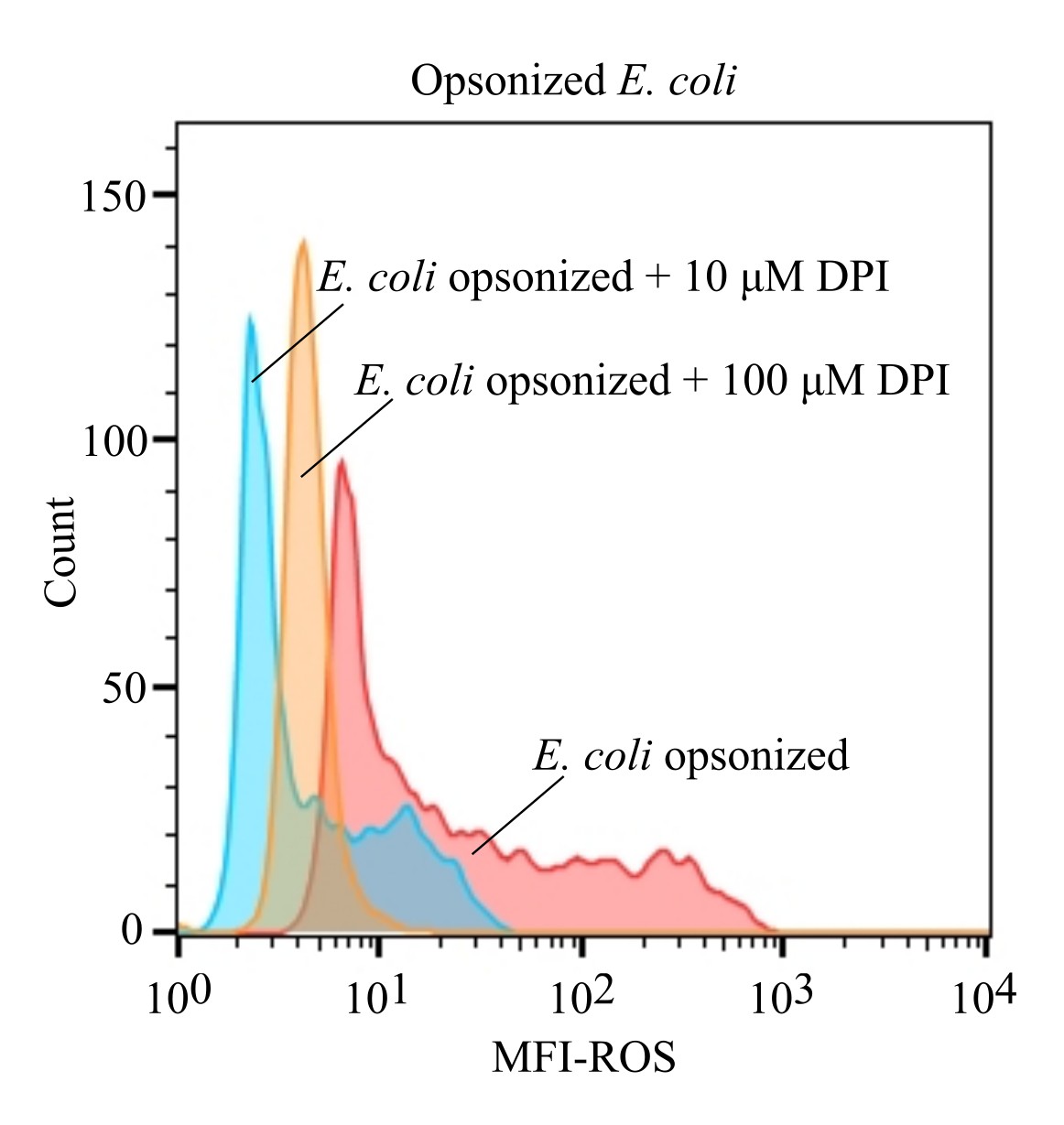

Supplement: Supplementary Figure 1 — HC neutrophils infected with opsonized E. coli bacteria in the absence and presence of 10 μM or 100 μM DPI. Data shown are representative of three independent experiments. [file Image1.jpeg]
